# Malaria parasite detection in thick blood smear microscopic images using modified YOLOV3 and YOLOV4 models

**DOI:** 10.1186/s12859-021-04036-4

**Published:** 2021-03-08

**Authors:** Fetulhak Abdurahman, Kinde Anlay Fante, Mohammed Aliy

**Affiliations:** 1 Faculty of Electrical and Computer Engineering, Jimma Institute of Technology, Jimma University, 378, Jimma, Ethiopia; 2 School of Biomedical Engineering, Jimma Institute of Technology, Jimma University, 378, Jimma, Ethiopia

**Keywords:** Malaria, *Plasmodium falciparum*, Thick blood smear, Deep learning, Object detection, YOLOV3, YOLOV4, Feature map

## Abstract

**Background:**

Manual microscopic examination of Leishman/Giemsa stained thin and thick blood smear is still the “gold standard” for malaria diagnosis. One of the drawbacks of this method is that its accuracy, consistency, and diagnosis speed depend on microscopists’ diagnostic and technical skills. It is difficult to get highly skilled microscopists in remote areas of developing countries. To alleviate this problem, in this paper, we propose to investigate state-of-the-art one-stage and two-stage object detection algorithms for automated malaria parasite screening from microscopic image of thick blood slides.

**Results:**

YOLOV3 and YOLOV4 models, which are state-of-the-art object detectors in accuracy and speed, are not optimized for detecting small objects such as malaria parasites in microscopic images. We modify these models by increasing feature scale and adding more detection layers to enhance their capability of detecting small objects without notably decreasing detection speed. We propose one modified YOLOV4 model, called YOLOV4-MOD and two modified models of YOLOV3, which are called YOLOV3-MOD1 and YOLOV3-MOD2. Besides, new anchor box sizes are generated using K-means clustering algorithm to exploit the potential of these models in small object detection. The performance of the modified YOLOV3 and YOLOV4 models were evaluated on a publicly available malaria dataset. These models have achieved state-of-the-art accuracy by exceeding performance of their original versions, Faster R-CNN, and SSD in terms of mean average precision (mAP), recall, precision, F1 score, and average IOU. YOLOV4-MOD has achieved the best detection accuracy among all the other models with a mAP of 96.32%. YOLOV3-MOD2 and YOLOV3-MOD1 have achieved mAP of 96.14% and 95.46%, respectively.

**Conclusions:**

The experimental results of this study demonstrate that performance of modified YOLOV3 and YOLOV4 models are highly promising for detecting malaria parasites from images captured by a smartphone camera over the microscope eyepiece. The proposed system is suitable for deployment in low-resource setting areas.

**Supplementary Information:**

The online version supplementary material available at 10.1186/s12859-021-04036-4.

## Background

Malaria is one of the leading public health burdens. Its prevalence is too high in low-income countries. In 2018 only, an estimated 228 million cases were recorded worldwide, and most of the cases were in Africa (comprising 93%), followed by South-East Asia region (3.4%). Malaria is caused by a unicellular parasite called plasmodium. It is transmitted from infected person to healthy through bites of female anopheles mosquito. Generally, there are five different species of plasmodium (*P. falciparum*, *P. vivax*, *P. ovale*, *P. malariae*, and *P. knowlesi*), of which *P. falciparum* is the most common, followed by *P. vivax*. Depending on the severity of infection, all the species pass through four life stages: ring, trophozoite, schizont, and gametocyte [1].

Malaria is a curable disease, but a lack of prompt and correct diagnosis and treatment can cause serious health complications, which can even lead to death. Microscopy-based visual examination of stained thick and thin blood slides is the golden standard for malaria diagnosis [2]. However, the accuracy of microscopy-based diagnosis heavily depends on individual’s slide reading experience and attentiveness during diagnosis procedure. Besides, shortage of well-trained personnel imposes discrepancies on the effectiveness of microscopy-based diagnosis results in malaria-endemic and resource-constrained areas, especially in rural parts of Africa. Inaccurate diagnosis result leads to morbidity, socio-economic problems within society and poor decision making and planning in malaria prevention programs.

An alternative low cost, fast and accurate computer-aided diagnosis system is required to overcome the drawbacks of manual microscopy-based diagnosis. Recent advancements in computer vision, especially in deep learning algorithms, have shown promising results for detecting malaria parasites and, in general, for detecting abnormalities in medical images.

For the past 2 decades, a substantial number of studies have been conducted to detect pathogens, including malaria parasites from microscopic images. The three most commonly used methods to detect or classify malaria parasites in microscopic images of thick and thin blood film are traditional image processing algorithms, classical machine learning, and deep learning method. The traditional image processing techniques mainly use rule-based classifiers and manually designed low-level features such as texture, color and shape using computationally complex image processing methods. The performance of these methods is low since manual design of optimal feature extractor and classifier is difficult [3–7].

Classical machine learning approaches, which use manually extracted features as inputs, were proposed for malaria parasite classification and detection in [8–12]. The proposed classifiers include support vector machine (SVM) [8], linear discriminant classification (LDC), k-nearest neighbor (KNN) and linear regression (LR) [5], Bayesian learning and support vector machine (SVM) [13], KNN classifier [9], hybrid machine learning classifier [10], modified K-means clustering algorithm [14, 15], feed-forward neural networks [6], deep belief network (DBN) [16]. The limitation of classical machine learning-based techniques is their inability to cope-up with inherent variability of images from different domains since manually designed feature extractor is sub-optimal. Machine learning models based on hand-crafted features achieve poor generalization capability in classification tasks.

In recent years, deep learning based object detection and classification techniques have gained popularity because of their ability to overcome the limitations of traditional image processing techniques, including classical machine learning algorithms. Deep learning algorithms can be applied to classify objects by taking image patches cropped from an input image using different prepossessing techniques such as segmentation or sliding window technique. These techniques are computationally expensive due to massive numbers of patches generated and the application of convolution operation for all patches to detect or classify malaria-infected cells [17, 18]. The methods in [19, 20] used features extracted from patch-based CNN and classifies them using classical machine learning models, such as SVM, for malaria parasite identification. Patch-based CNN models for the identification of malaria parasites from thick blood smear microscopic images were proposed by [21–24]. The studies by [25–27] demonstrated the applicability of patch based CNN models to classify malaria parasites from segmented thin blood smear images. Sivaramakrishnan et al. [28] discussed advantage of visualizing extracted features using deep learning models to better understand their learning strategy for classification of malaria parasites. Delahunt et al. [29] proposed a patient-level evaluation results using two different CNN architectures to detect ring and late-stage malaria parasites.

Lately, state of the art one-stage and two-stage object detection algorithms are widely used in diverse applications of medical image analysis including detection of cancer [30–33], detection of organs and their abnormalities [34–37], detection of pulmonary diseases [38–40], detection and segmentation of intracranial hemorrhages [41], classification and segmentation of microscopy images [35, 42, 43].

Despite their success in several applications, state-of-the-art deep learning-based one-stage and two-stage object detection algorithms have not been extensively studied to detect malaria parasites in microscopic images. The work reported in [44] uses a modified YOLOv3 architecture to detect *P. falciparum* parasites in thick blood smear microscopic images taken with a digital microscope and smartphone camera. An automated *P.vivax* detection system in microscopic images of thin blood smear was reported in [45]. A pre-trained Faster RCNN model was applied to classify red blood cells (RBCs) and other non-RBC objects [46].

This study aims to investigate the applicability of state-of-the-art one-stage and two-stage object detection algorithms for detecting malaria parasites in microscopic images captured using a smartphone camera. We propose malaria parasite detection in thick blood smear microscopic images using modified YOLOV3 and YOLOV4 models in this work. The contributions of this study are as follows:

1. We have modified YOLOV3 and YOLOV4 models to improve their capability to detect small objects by extending feature scales and adding more detection layers. The modified models have higher small object detection capabilities than the original models.
2. We have conducted comprehensive experiments to evaluate performance of the original and modified models of YOLOV3 and YOLOV4 using publicly available malaria dataset.
3. We have also carried out a comprehensive comparative study to evaluate the performance of state of the art two-stage and one-stage object detection algorithms such as Faster RCNN [47], SSD [48], YOLOV3 [49], and YOLOV4 [50] to detect malaria parasites.

## Results

In this study, we have conducted experiments to evaluate the performance of state-of-the-art one-stage and two-stage deep learning-based object detectors for detecting *P. falciparum* in thick blood smear microscopic images captured using a smartphone camera. Performance evaluations of the proposed models, using mean average precision (mAP), precision, recall, F1 score, average IOU and inference time in frames per second (FPS), are done at object-level.

### Performance analysis of modified YOLOV3 and YOLOV4 models

**Fig. 1:**
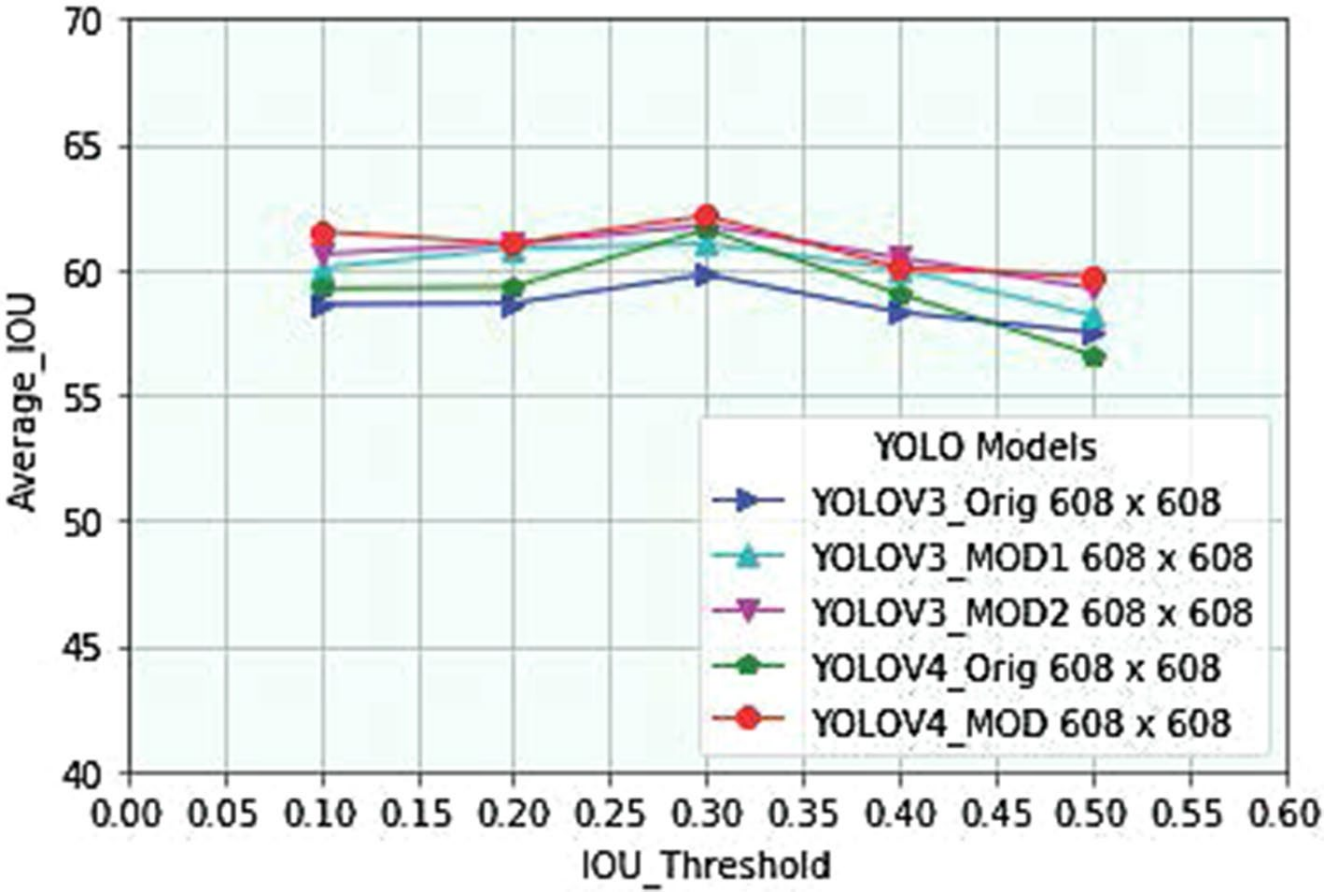
Analysis of IOU threshold values for optimal localization of *P. falciparum*

**Fig. 2:**
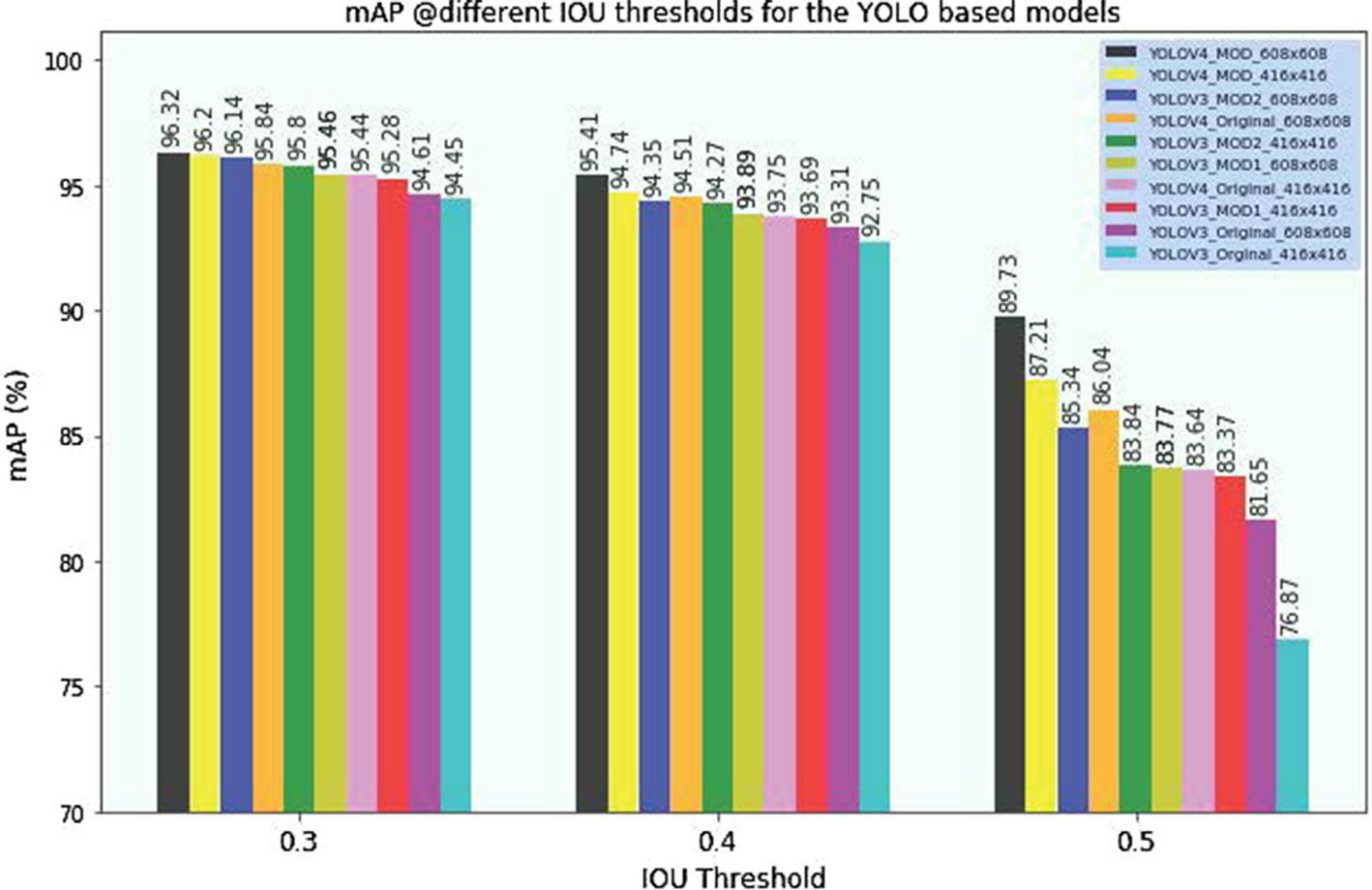
The effect of IOU threshold values on performance of YOLO-based models during inference time

For YOLO-based models (YOLOV3, YOLOV4, and their modified versions), we have used an IOU threshold value of 0.3, which is selected experimentally by analyzing average IOU using validation dataset. As depicted in Fig. 1, YOLO-based models have achieved the highest average IOU value at a threshold value of 0.3. When we increase the IOU threshold value above 0.3, average IOU of these models decreases since only a small number of tight bounding boxes are predicted enclosing malaria parasites. Similarly, when we adjust the threshold value below 0.3, the models’ average IOU decreases due to the prediction of many loose bounding boxes that enclose malaria parasites. Therefore, we have selected an IOU threshold value of 0.3 to evaluate performance of our models during inference time. The proposed models’ performance at different IOU threshold values during inference is shown in Fig. 2, indicating that malaria parasite detection results are sensitive to predefined IOU threshold values.

Our experimental results depicted in Table 1 illustrate that YOLOV4-MOD achieves the highest *P. falciparum* detection accuracy with mAP of 96.32% and 96.20% for input image resolutions of $608 \times 608$ and $416 \times 416$, respectively. The original YOLOV4 achieves mAP of 95.84% and 95.44% for $608 \times 608$ and $416 \times 416$ input image resolutions respectively. All YOLO-based models have achieved high *P. falciparum* detection performance for an input image resolution of $608 \times 608$. When the input image resolution is reduced to $416 \times 416$, the models’ performance slightly decreases and detection speed increases. YOLOV3-MOD2, with an input image resolution of $608 \times 608$, has achieved the third rank, with a mAP of 96.14%, compared to other YOLO-based models, and it is best performing model among YOLOV3-based models. It also has a balanced precision (92%) and recall (93%) rate indicating that it can discriminate well artifacts from malaria parasites. YOLOV3-MOD1 has achieved mAP of 95.46% and 95.28% for input image resolution of $608 \times 608$ and $416 \times 416$, respectively. YOLOV3- MOD1 has achieved lower detection performance than YOLOV3-MOD2, but it still has a higher performance compared to the original YOLOV3 model to detect malaria parasites. Overall, YOLOV4-MOD is the best performing model in terms of mAP, precision (95%), recall (94%) and F1-score (94%) on our test dataset.

Besides, average IOU values of different models indicate their ability for precise localization of malaria parasites during inference time. Table 1 shows that the modified YOLOV3 and YOLOV4 models have achieved higher average IOU values than their original counterparts. The detection speed comparison of different models during inference time is also shown in Table 1, represented by frames per second (FPS). As shown in the table, YOLOV4-MOD has a slightly lower *P. falciparum* detection speed than its original version at inference time. Inference time *P. falciparum* detection speed of YOLOV3-MOD2 is also less than its original version. This is due to high computational complexity of convolution operation at shallow feature maps with a large feature scale in YOLOV3-MOD2.

## Discussion

In general, YOLOV4 models have achieved better *P. falciparum* detection performance than YOLOV3 models in both their original and modified models. YOLOV4-MOD is the best model for *P. falciparum* detection among all YOLO-based models. Our modified YOLOV3 and YOLOV4 models, with fine-grained features at high-resolution feature maps, have achieved better detection performance compared with their original versions for small object detection, such as *P. falciparum*. This implies that the modified network structures learn more robust geometric and semantic information to discern small objects than their original versions.

### Comparative analysis of modified YOLOV3 and YOLOV4 models with existing methods

Performance comparison of modified YOLOV3 and YOLOV4 models with recent state-of-the-art one-stage detector SSD and two-stage detector Faster R-CNN is shown in Table 1. As shown in the table, YOLO-based models, both modified and original versions, have achieved considerably higher *P. falciparum* detection performance than Faster R-CNN and SSD models. Faster R-CNN has achieved 71.0% mAP while SSD has achieved a mAP of 71.4%. The SSD model has the highest *P. falciparum* detection speed, in frames per second, among all the other models, but it has worst detection accuracy. Faster R-CNN model is the slowest in its detection speed, and its detection accuracy is comparable to that of the SSD model.

The performance of our modified YOLO-based malaria parasite detection models is also compared with existing related works. Chibuta and Acar [44] proposed a modified YOLOV3 model to detect malaria parasites using the same dataset as that of this work. Their model achieved a mAP of 90.2%, which is lower than the performance of all three modified YOLO-based models proposed in this work, where YOLOV4-MOD has achieved a mAP of 96.32 %. Their model detection computation time was measured in CPU time, which is 0.42 s per image, whereas our best performing model has a computation time of 0.034 s per image measured in GPU time. Another method proposed by authors of [45] used a cascade of YOLOV2 and transferred AlexNet and achieved a mAP of 79.22%, which is significantly lower than our models’ performance. Hung et al. [46] reported a 98% of accuracy using Faster R-CNN in cascade with AlexNet to identify *P.vivax*. Their model’s reported performance outperforms our best performing (YOLOV4-MOD) model, which has an accuracy of 94.36%. However, Hung et al. [46] used thin blood sample microscopic images in which parasitic objects are bigger than the ones in thick blood samples, and small object detection is more challenging than large object detection in images. The cascaded Faster R-CNN with AlexNet model is computationally expensive due to its two-stage architecture and additional AlexNet component. The datasets used in [45, 46] are not the same as ours.

**Table 1:** Performance comparison of proposed *P. falciparum* detection models using test dataset

Models	mAP@0.3 (%)	Precision (%)	Recall (%)	F1-Score (%)	Avg. IOU (%)	FPS
YOLOV4-MOD @$608 \times 608$	**96.32**	**95**	**94**	**94**	**62.12**	**29.60**
YOLOV4-MOD @$416 \times 416$	**96.20**	**93**	**93**	**93**	**61.84**	**30.56**
YOLOV3-MOD2 @$608\times 608$	96.14	92	93	92	61.77	15.30
YOLOV3-MOD2 @$416\times 416$	95.80	92	92	92	61.03	17.83
YOLOV3-MOD1 @$608\times 608$	95.46	92	92	92	61.03	21.40
YOLOV3-MOD1 @$416\times 416$	95.28	92	92	92	60.64	26.75
YOLOV4 @$608\times 608$ [50]	95.84	92	92	92	61.15	30.77
YOLOV4 @$416\times 416$ [50]	95.44	92	92	92	60.67	33.89
YOLOV3 @$608\times 608$ [49]	94.61	91	92	92	59.98	28.67
YOLOV3 @$416 \times 416$ [49]	94.45	91	91	91	58.85	30.43
Faster R-CNN [47]	71.0	92.7	86.9	89.71	–	8
SSD @$300\times 300$ [48]	71.4	91	84	87	–	41

Bold values indicate best performing models

### Visual analysis of test results

**Fig. 3:**
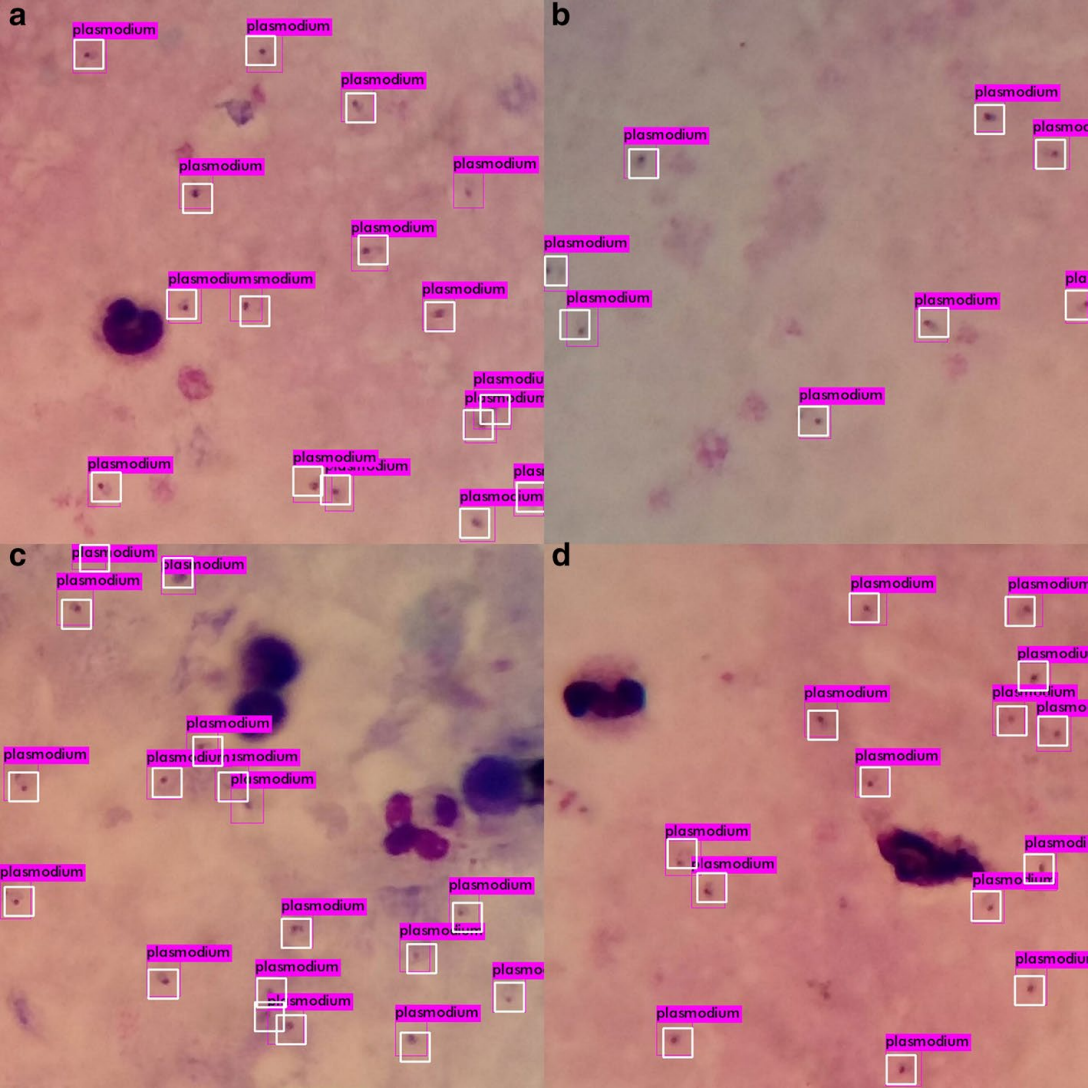
Example *P. falciparum* detection results using YOLOV4-MOD @$608\times 608$

To further evaluate *P. falciparum* detection performance of different models used in this study, example detection results for four images taken from our test dataset is presented in Fig. 3 for visual analysis. In Fig. 3, we can observe that YOLOV4-MOD has detected all parasites with only one false positive case in the first image. Similarly, it has correctly detected all parasites in the third image except for one false negative and one false positive predictions. The ground truth and predicted bounding boxes are shown in white and violet colors, respectively. For the remaining models, examples are provided as a supplementary file (see Additional file 1). For visual analysis of malaria parasite detection results, we have used $608\times 608$ input image resolution. In general, from the visual analysis of detection results, we can see that the modified YOLOV3 and YOLOV4 models’ malaria parasite detection performance is better than that of other models with good localization accuracy.

## Conclusions

In this paper, performances of state-of-the-art deep learning based object detection algorithms are thoroughly investigated for malaria parasite detection in thick blood smear microscopic images. We have modified YOLOV3 and YOLOV4 network architectures to enhance their performance for small object detection task. Several experiments are conducted to compare performance of our modified YOLOV3 and YOLOV4 based models with existing models such as SSD and Faster R-CNN. YOLOV4-MOD has outperformed all the other models with a mAP of 96.32% for $608\times 608$ input image resolution. Similarly, YOLOV3-MOD2 and YOLOV3-MOD1 have achieved a mAP of 96.14% and 95.46% for the same input image resolution, respectively. The proposed models outperform their original versions, Faster R-CNN and SSD models in terms of mean average precision (mAP), recall, precision, F1 score, average IOU and speed of object detection. We have demonstrated the feasibility and effectiveness of proposed YOLOV-based architectures for *P. falciparum* detection in microscopic images captured using a smartphone camera. Our future work will investigate the applicability of these algorithms for detection of various plasmodium species and stages both in thin and thick blood smear microscopic images. The reliability of these algorithms will also be studied in our future work.

## Methods

To improve small object detection accuracy of YOLOV3 and YOLOV4 models, we have modified these network architectures by including more fine-grained features from low-resolution feature maps. The performance of proposed algorithms, in terms of detection speed and accuracy, have been investigated for detection of *P. falciparum* using microscopic images taken using a smartphone camera.

### Dataset

**Fig. 4:**
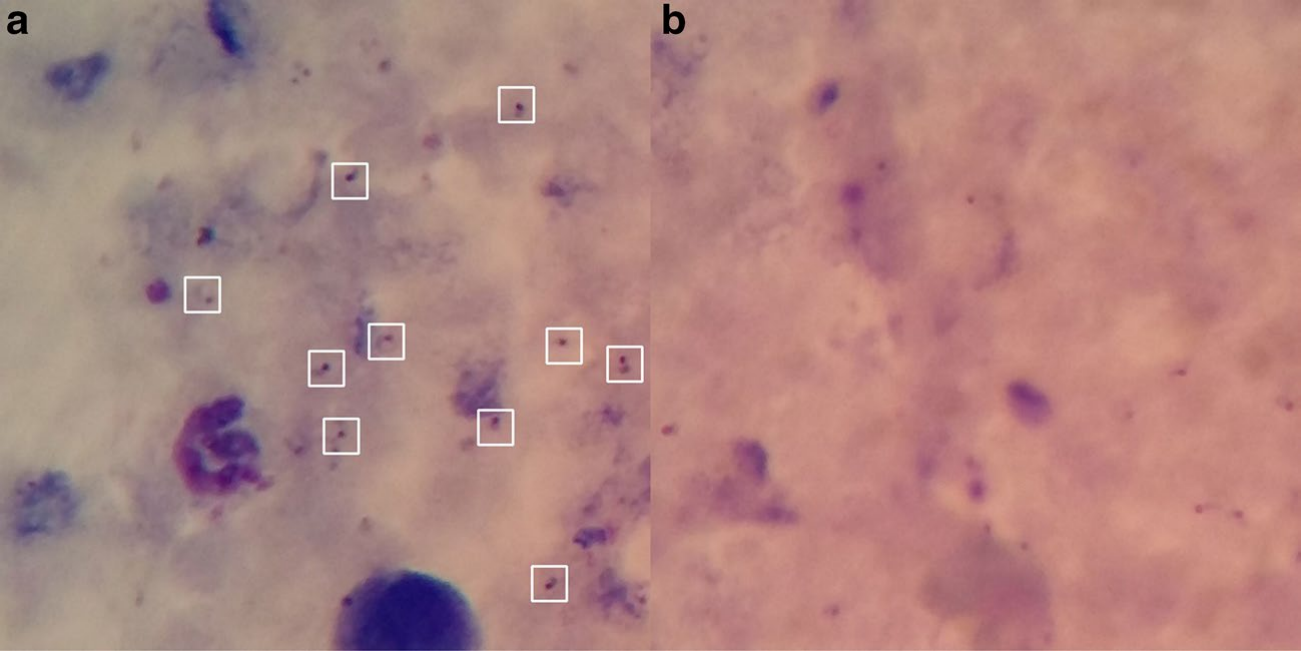
Sample negative (right) and positive (left) microscopic images with ground truth bounding box *P. falciparum* locations

We used publicly available malaria dataset [23]^1^ for the analysis presented in this study. It was collected using a smartphone camera attached to a microscope’s eyepiece using a special attachment device developed for this purpose. The dataset contains 1182 color microscopic images of thick blood smear malaria slides, which were stained with Field stain at x1000 magnification level, and all the images have a resolution of $750\times 750$ pixels. It contains 948 malaria-infected images with 7628 *P. falciparum* parasites and 234 normal images with artifacts due to impurities. Since the dataset consists of only *P. falciparum*, our models were trained only to detect this malaria parasite species. All malaria parasite detection models in this study were trained using 90% of the dataset, among which we took 10% of it for validation. The remaining 10% of the dataset was used to test performance of *P. falciparum* detection models. Figure 4 shows examples of malaria-infected (positive) and normal (negative) microscopic images from our dataset and corresponding bounding box locations of *P. falciparum*. Detail description of images in training, validation and testing data sets is listed in Table 2. The images were annotated by expert laboratory technicians. No additional patient-level information is given in the dataset, such as the number field of views taken for a single patient. Thus, we have evaluated performance of proposed models at parasite level.

**Table 2:** Detail descriptions of malaria dataset used in this study

	Training	Validation	Testing	Total
Number of images	966	109	107	1182
Negative samples	191	23	20	234
Positive samples	775	86	87	948
Number of parasites	5695	924	1009	7628

### Malaria parasite detection architectures

YOLOV3 [49] is one of the most potent single-stage object detection algorithms used in various application areas. It remarkably improves object detection accuracy, object bounding box localization and speed of detection compared with its previous versions; YOLOV1 [51] and YOLOV2 [52]. The network structure of YOLOV3 is modeled as a single regression problem having one backbone CNN and three object detection heads known as yolo layers. These three detection heads divide an input image into three different grids of size S $\times$  S. Each grid cell is responsible for detecting objects whose center falls on that grid cell. In YOLOV3 model with an input image resolution of $416\times 416$, the feature map size is $13 \times 13, 26 \times 26$, and $52 \times 52$ for the first, second and third detection layers, respectively.

**Fig. 5:**
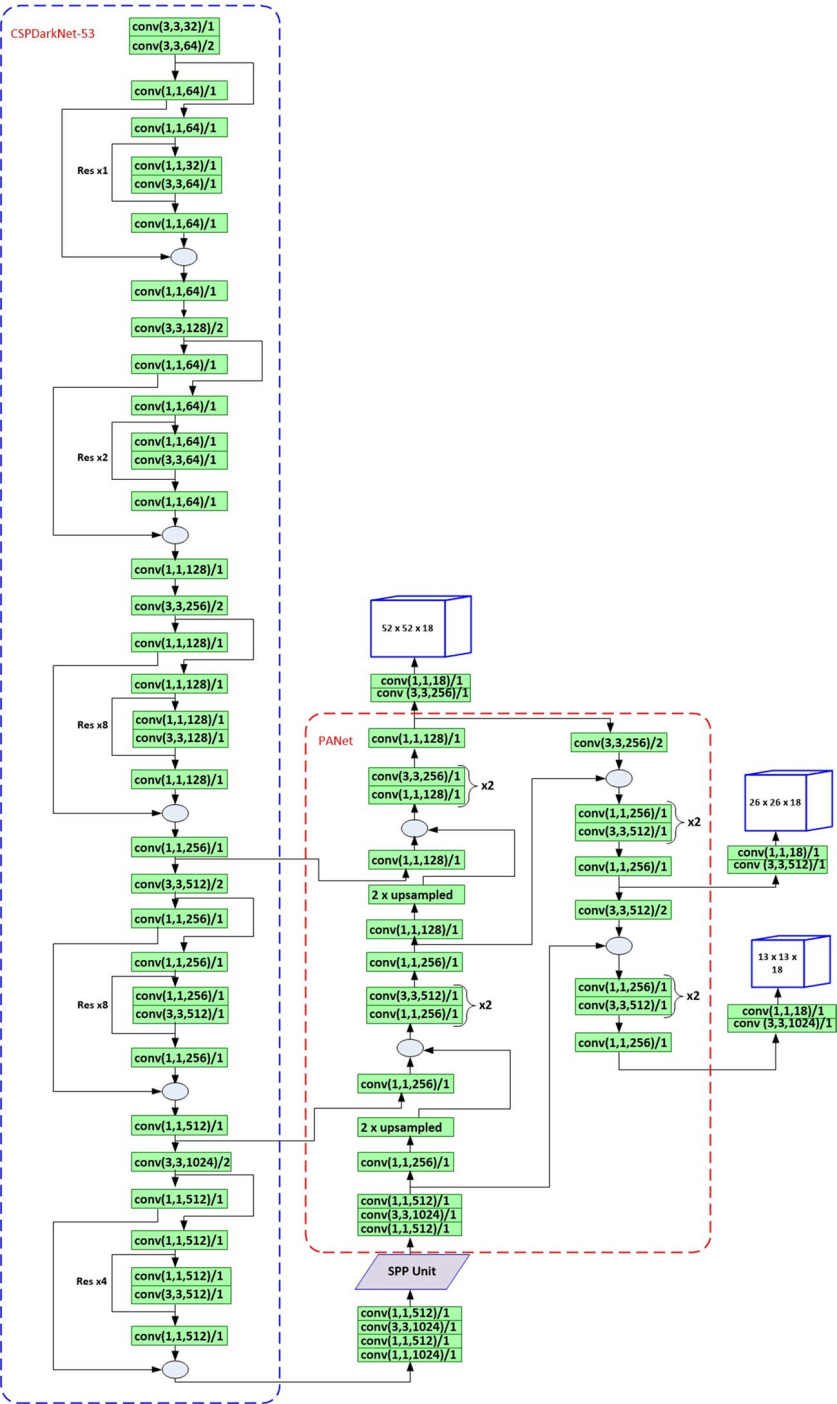
Network architecture of YOLOV4

YOLOV4 model [50] is a state-of-the-art deep learning-based object detection technique with superior performance compared to other recent models such as EfficientDet [53] and YOLOV3 [49]. Authors of [50] combined different features from other studies, which has enhanced performance of YOLOV4 with a low computation cost during inference. The features added in YOLOV4 architecture are categorized into two methods called Bag of Freebies(BOF) and Bag of Specials(BOS). The BOF and BOS are applied both in the backbone and detector modules of YOLOV4 architecture. In the BOS part, spatial pyramid pooling (SPP) is tightly coupled with the backbone network to improve receptive field sizes of detection layer feature maps. BOF mostly comes from data augmentation techniques such as Mosaic, CutMix, and self-adversarial training (SAT). The detection heads of YOLOV4 are similar to YOLOV3 with three detection feature maps, which are generated through a concatenation of feature maps at different levels of convolution operation. Figure 5 shows the detailed network architecture of YOLOV4.

#### Modified YOLOV3 and YOLOV4 architectures

The original models of YOLOV3 and YOLOV4 were trained and evaluated using Imagenet, Pascal VOC [54], and MS COCO [55] datasets. These datasets contain natural images with objects far bigger than malaria parasites in microscopic images. The original models of YOLOV3 and YOLOV4, without modifying network architectures and hyperparameter optimization, achieve low performance in detecting small objects such as malaria parasites.

**Fig. 6:**
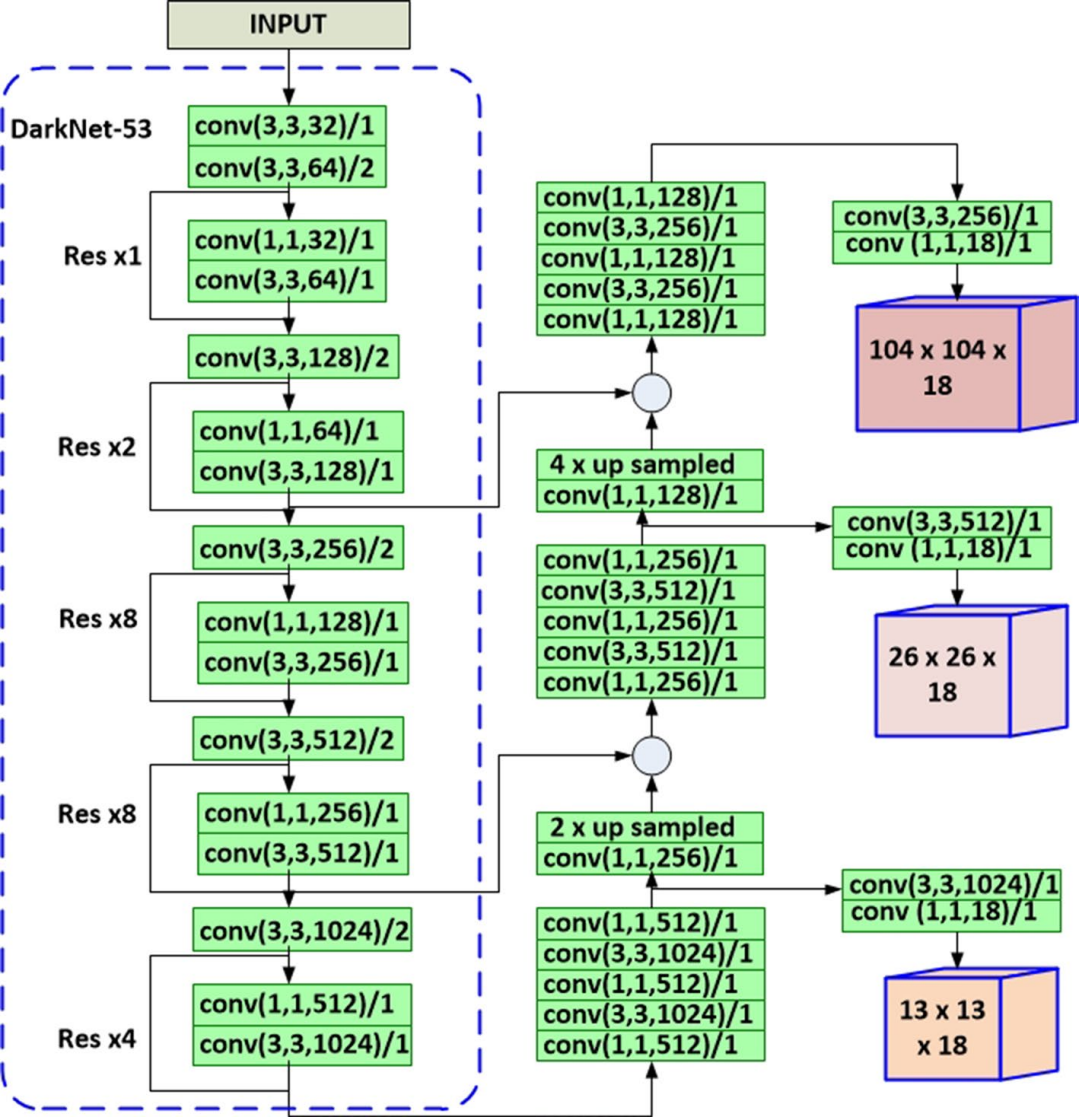
Architecture of YOLOV3-MOD1

In this study, we have modified the original YOLOV3 network architecture to obtain two different architectures, which we call YOLOV3-MOD1 and YOLOV3-MOD2. In YOLOV3-MOD1, we have changed the shallow feature map scale to $104 \times 104$, which improves the detection of *P. falciparum* compared with the original YOLOV3 model, which has bigger receptive fields than the *P. falciparum* size. Figure 6 shows the network structure of YOLOV3-MOD1. As shown in the figure, the modified YOLOV3-MOD1 has detection layer feature maps of size $13 \times 13, 26 \times 26$, and $104 \times 104$. This modified multi-scale feature map extracts more robust features for *P. falciparum* detection with a minimal added computational cost.

**Fig. 7:**
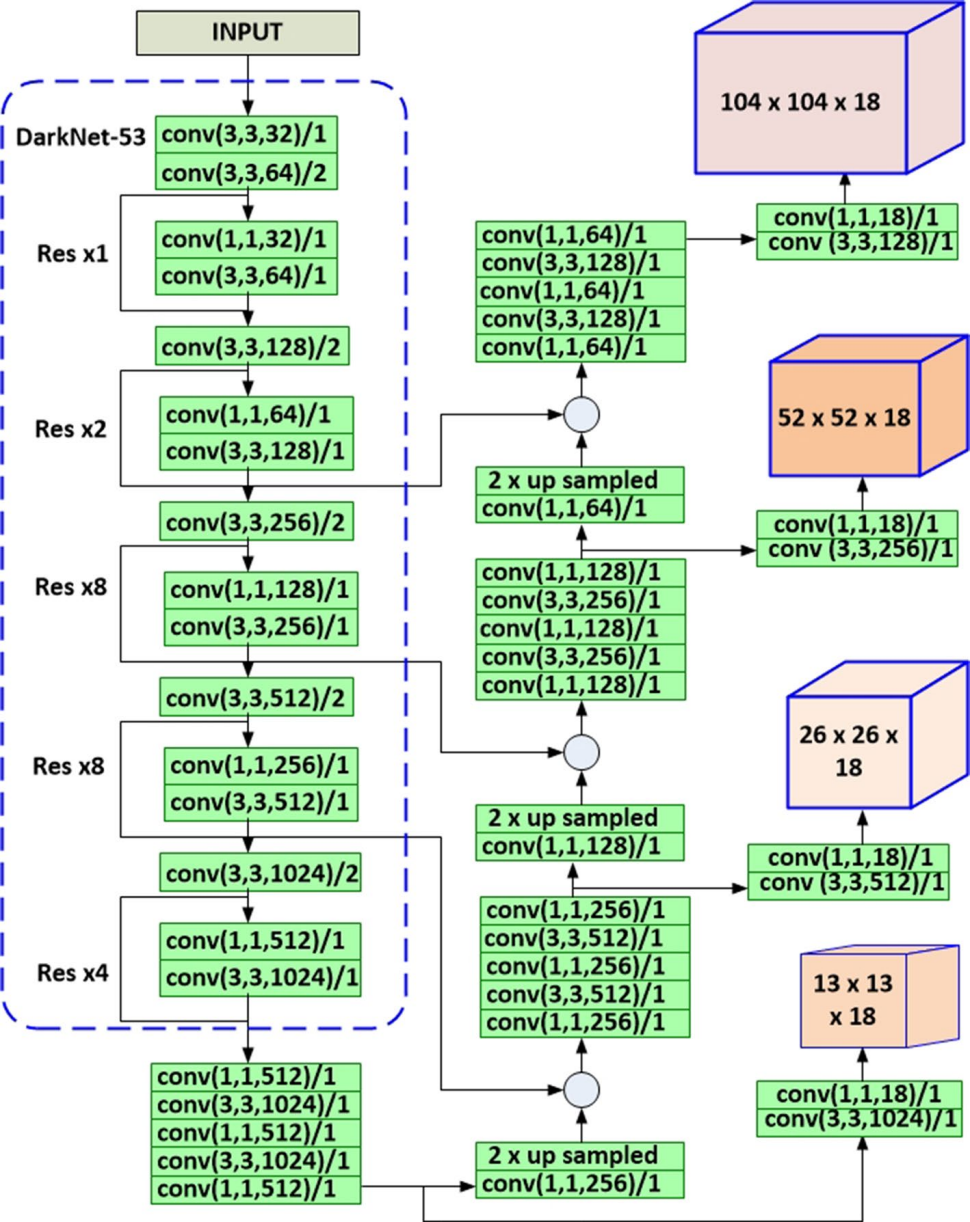
Architecture of YOLOV3-MOD2

In YOLOV3-MOD2, we have added a fourth detection layer into the existing three detection layers of the original YOLOV3 model. The added layer increases the performance of YOLOV3 for small object detection since a short connection of deeper features with shallow features enhances the fine-grained feature discerning ability of the detection layers. The proposed YOLOV3-MOD2 model is shown in Fig. 7. It has four detection feature maps with size $13\times 13, 26 \times 26, 52 \times 52$, and $104 \times 104$. We have also added three additional anchor box sizes for the newly added detection layer. We have used K-means clustering algorithm to generate nine new anchor box sizes for YOLOV3-MOD1, and 12 new anchor box sizes for YOLOV3-MOD2 based on ground truth bounding box sizes from our malaria dataset. These two modified YOLOV3 models improve the performance of *P. falciparum* detection compared to the original YOLOV3 model. Performance analysis of these models is given in detail in “Results” section. YOLOV3-MOD2 has better detection accuracy than YOLOV3-MOD1 but at the cost of reduced detection speed during inference time.

Similarly, a modified YOLOV4 model, which is called YOLOV4-MOD hereafter, is obtained by adding a fourth detection layer into the existing YOLOV4 network architecture. The added layer enables the YOLOV4-MOD model to obtain robust geometric features concatenated with deeper level features using PANet architecture [56]. By adding this layer, we can obtain more comprehensive features that enhance the performance of YOLOV4-MOD for small object detection. For this network architecture, we have generated 12 new anchor box sizes, which are evenly distributed to the four detection layers based on their size. YOLOV4-MOD is found to be the best performing model for *P. falciparum* detection in our experiments.

#### Training and hyper-parameter optimization

In the training phase of the proposed malaria parasite detection models, we have used a pre-trained weight for each detection model by experimentally selecting the best pre-trained weight using our malaria dataset. We have then re-trained the models to adapt to our malaria parasite detection task by fine-tuning these weights.

We have used Darknet Framework to train YOLO-based models (YOLOV3, YOLOV4, YOLOV3- MOD1, YOLOV3-MOD2, and YOLOV4-MOD). The training of these models was performed for 4000 iterations using stochastic gradient descent (SGD) method with Adam optimization algorithm. We have used an initial learning rate of 0.001, a batch size of 64 with subdivision 32, and a momentum of 0.9 with a weight decay of 0.0005. The learning rate was reduced by multiplying it by 0.1 at 3200 and 3600 training iterations. Pre-trained weight files using ImageNet and MS COCO [55] data sets were fine-tuned using our malaria dataset for YOLOV3 and YOLOV4 based models, respectively. We have trained YOLO-based models using input image sizes of $416 \times 416$ and $608 \times 608$. During the training phase of these models, multi-scale training was enabled by changing the input resolution in the range $(320 \times 320)$ to ($896 \times 896)$ every ten training iterations on the fly. This enables YOLO-based models to detect objects at different image resolutions. We have used modified anchor box sizes generated using K-means clustering algorithms based on ground truth bounding box sizes obtained from our malaria dataset. For YOLOV3, YOLOV4, and YOLOV3-MOD1 models, we have used nine new anchor box sizes. Similarly, for YOLOV3-MOD2 and YOLOV4-MOD models, we have generated 12 new anchor box sizes which are evenly distributed to detection layers as per their size.

Another hyper-parameter that has been optimized is a threshold value for intersection over union (IOU), which is object detection model evaluation metrics. The IOU quantifies how much the ground truth annotation of an object overlaps with its predicted bounding box by a model. It is given by the following equation.

1 $$\begin{aligned} IOU(A,B) = \frac{A \cap B }{A \cup B} \end{aligned}$$

where A is the ground truth box of an object, and B is predicted bounding box by object detection model. The IOU value is compared with a pre-defined hyper-parameter called IOU threshold to determine whether the predicted bounding box is a true positive class (i.e., in our study *P. falciparum*) or a false positive. If the IOU value is greater than the threshold value, the predicted bounding box is classified as true positive (*P. falciparum*); otherwise, it is classified as false positive. The selection of IOU threshold value affects the mean average precision value of all the studied models. Therefore, selecting an optimal IOU threshold value for *P. falciparum* detection is necessary to obtain the best possible results in terms of true positives and false positives. We have experimentally selected an optimal IOU threshold value of 0.3 in this study.

We have also tuned different hyper-parameters of Faster R-CNN and SSD models empirically based on their default configuration in Tensorflow Object detection API. We have experimentally selected Inception-v2 as a feature extractor for both Faster R-CNN and SSD models, which is pre-trained on MS COCO dataset. For Faster R-CNN, we have selected aspect ratios of 1:1, 1:2 and 2:1, and scales of [0.1, 0.15, 0.2, 0.25] since the default anchor box sizes are much bigger than the size of malaria parasite in microscopic images. We fine-tuned a pre-trained Faster R-CNN model using our malaria dataset with an initial learning rate of 0.001, which is reduced by 0.1 factor at training iterations of 65,000 and 85,000 and optimized using momentum SGD. We trained Faster R-CNN model for a total of 100K iterations. For SSD model, we have used a modified minimum scale of 0.1 and a maximum scale of 0.9 to generate suitable anchor box sizes for our malaria dataset. We trained SSD with $300 \times 300$ input image size. We have carried out additional experiments to select optimal initial learning rate of 0.001 with a batch size of 24, and RMSProp optimizer to train the SSD model for 100K iterations. For both SSD and Faster R-CNN models, we adopted a drop out unit with a value of 0.5 to overcome the problem of over-fitting. The experiments were conducted on Google Colab cloud service with Tesla T4 GPU and 12 GB GDDR5 VRAM for all *P. falciparum* detection models.

## Supplementary Information

**Additional file 1.** Examples of malaria parasite detection results using different detection models.

## Data Availability

The dataset used during the current study is available online at http://air.ug/downloads/plasmodium-phonecamera.zip [23].

## Abbreviations

YOLOV3: You Only Look Once Version 3
YOLOV4: You Only Look Once Version 4
mAP: Mean average precision
SVM: Support vector machine
LDC: Linear discriminant classification
KNN: K-nearest neighbor
DBN: Deep belief network
CNN: Convolutional neural network
RCNN: Region based convolutional neural network
RBC: Red blood cell
SSD: Single shot multibox detector
FPS: Frames per second
IOU: Intersection over union
GPU: Graphics processing unit
RGB: Red, green and blue
BOF: Bag of freebies
BOS: Bag of specials
SAT: Self-adversarial training
PASCAL: Pattern analysis, statistical modeling and computational learning
VOC: Visual object classes
MS: Microsoft
COCO: Common objects in context
MOD: Modified
PANet: Path aggregation network
SGD: Stochastic gradient descent
API: Application programming interface
GDDR5: Graphics double data rate 5
VRAM: Video random access memory

## Footnotes

1 http://air.ug/downloads/plasmodium-phonecamera.zip.
