# Supplementary material for: Malaria parasite detection in thick blood smear microscopic images using modified YOLOV3 and YOLOV4 models
Source: BMC Bioinformatics. 2021 Mar 8;22:112. doi: 10.1186/s12859-021-04036-4 (PMC7938584; doi:10.1186/s12859-021-04036-4)
Supplement: Supplementary file 1 — Additional file 1. Examples of malaria parasite detection results using different detection models. [file 12859_2021_4036_MOESM1_ESM.pdf]

1 Visualization Test Results

To further evaluate the *p. falcuparum* detection performance of the different detection models used in this study partial detection results of the models are shown in Figures 1-7. In the figures, we have used the 608 x 608 resolution for the YOLO based models since all the models achieve better detection results at this resolution. The figures indicate the performance of the different models under the same four test images. The ground truth and the predicted bounding boxes are shown in white and violet respectively. As shown from Figure 1 the YOLOV4-MOD detects all the parasites with only one false positive case in the first image and one false negative and one false positive prediction results in the third image. The YOLOV4-MOD has shown better detection results compared with the original YOLOV4 shown in Figure 4 with minimal added computation cost. This is mainly due to rich feature information learned by concatenating additional shallow level features with semantic rich high-level features in the modified YOLOV4 architecture.

In Figures 2 and 3 the YOLOV3-MOD2 and YOLOV3-MOD1 models correctly recognize the *p.falciparum* with only few incorrectly detected parasites compared with the original YOLOV3 in Figure 5 which misses more parasites. For the Faster R-CNN and SSD models as shown in Figures 6 and 7 the models predicts a lot of parasites as non-parasite as compared to the modified models. In general, from the figures we can see that the detection performance of the improved YOLOV3 and YOLOV4 models shown better detection results with good localization as compared to their original versions and other detection models.

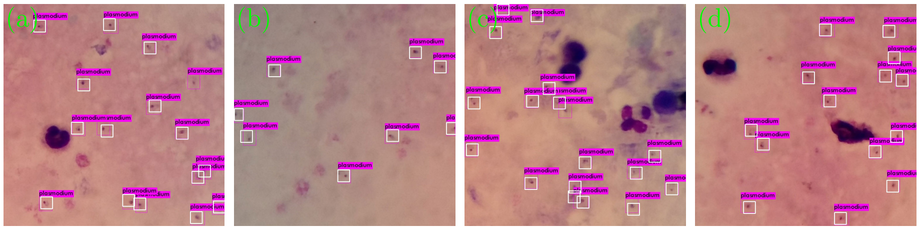

Figure 1: YOLOV4-MOD @608 x 608

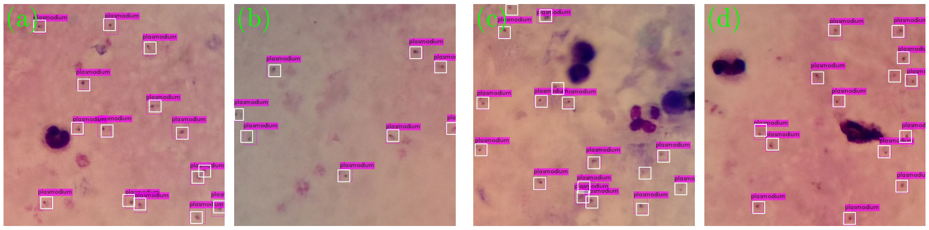

Figure 2: YOLOV3-MOD2 @608 x 608

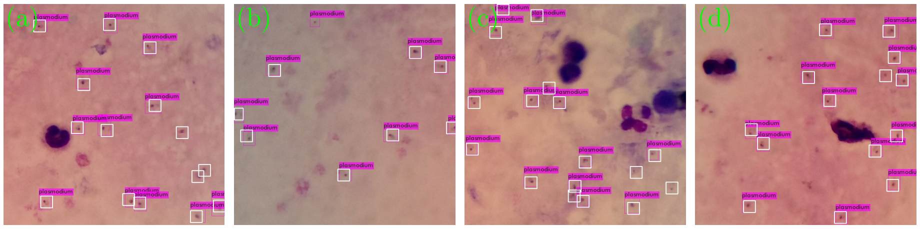

Figure 3: YOLOV3-MOD1 @608 x 608

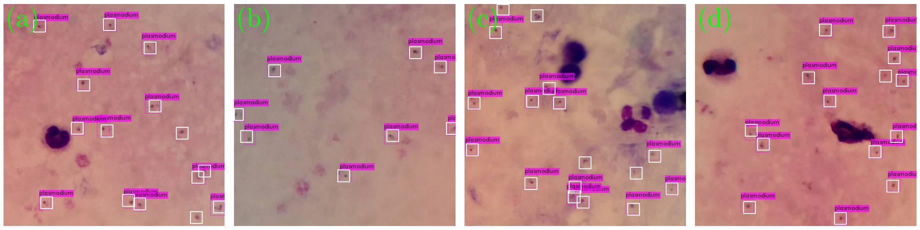

Figure 4: YOLOV4-Original @608 x 608

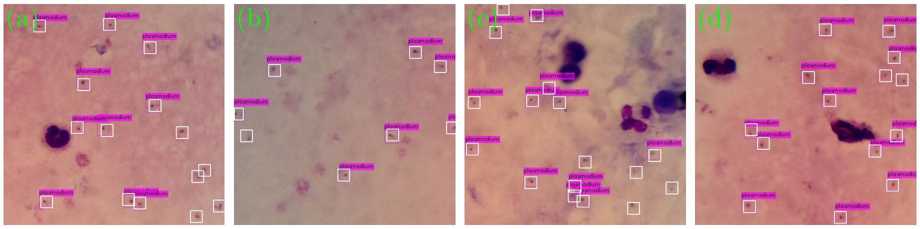

Figure 5: YOLOV3-Original @608 x 608

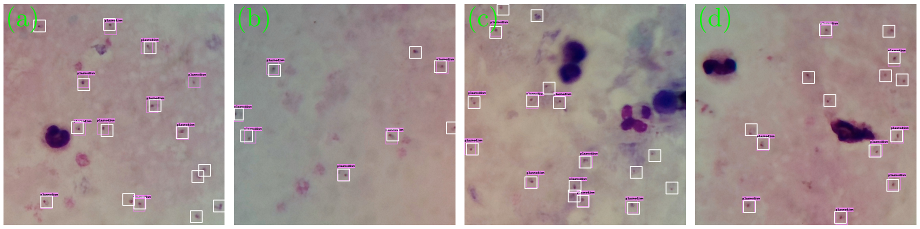

Figure 6: SSD @300 x 300

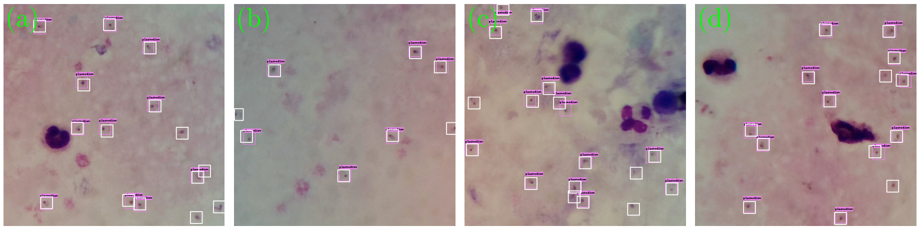

Figure 7: Faster R-CNN
